# Triggers for Atrial Fibrillation: The Role of Anxiety

**DOI:** 10.1155/2019/1208505

**Published:** 2019-02-18

**Authors:** Paolo Severino, Marco Valerio Mariani, Annalisa Maraone, Agostino Piro, Andrea Ceccacci, Lorenzo Tarsitani, Viviana Maestrini, Massimo Mancone, Carlo Lavalle, Massimo Pasquini, Francesco Fedele

**Affiliations:** ^1^Department of Cardiovascular, Respiratory, Anesthesiology, Nephrology and Geriatric Sciences, Sapienza University of Rome, Rome, Italy; ^2^Department of Human Neurosciences, Sapienza University of Rome, Rome, Italy; ^3^Department of Neurosciences and Mental Health, Umberto I Policlinic, Rome, Italy

## Abstract

Atrial fibrillation (AF) is the most widely recognized arrhythmia. Systemic arterial hypertension, diabetes, obesity, heart failure, and valvular heart diseases are major risk factors for the onset and progression of AF. Various studies have emphasized the augmented anxiety rate among AF patients due to the poor quality of life; however, little information is known about the possibility of triggering atrial fibrillation by anxiety. The present review sought to underline the possible pathophysiological association between AF and anxiety disorders and suggests that anxiety can be an independent risk factor for AF, acting as a trigger, creating an arrhythmogenic substrate, and modulating the autonomic nervous system. The awareness of the role of anxiety disorders as a risk factor for AF may lead to the development of new clinical strategies for the management of AF.

## 1. Introduction

Atrial fibrillation (AF) is the most common arrhythmia in clinical practice, with an overall prevalence of 1-2% in the general population [1] and an incidence that increases with age up to 20% in octogenarians. In the next 50 years, its prevalence is expected to double, as a consequence of the prolongation of life expectancy [2]. Five types of AF are classified: first diagnosed, paroxysmal, persistent, long-standing persistent, and permanent AF (KirchhoffAF and psychological factors). AF is associated with high relative risk of all-cause mortality, stroke, cardiovascular mortality, cardiac events, heart failure, and chronic cognitive impairment and represents the most common arrhythmia that requires hospitalization and one of the most frequent causes of hospitalization for heart diseases. Several risk factors and heart diseases are known to be involved in the genesis and/or perpetuation of AF, acting by different pathophysiological pathways on the presence of a susceptible atrial electroanatomic substrate. Among risk factors [3], both unmodifiable, as genetic susceptibility, age, gender, race, and modifiable, as systemic arterial hypertension, diabetes mellitus, smoking, obstructive sleep apnea, and obesity, have adverse effects on cardiovascular hemodynamic as well as on cardiac structure and function, increasing the prevalence of AF. Moreover, heart failure (HF) and AF frequently coexist: HF predisposes AF and vice versa. Left ventricular dysfunction, independently from ejection fraction, is linked to AF by a hemodynamic overload [4]; on the other hand, as well known, AF can decrease overall cardiac output from loss of atrial kick [5].

Over the last decades, epidemiological studies associated various risk factors with AF. Moreover, many reports have advanced the hypothesis of a mutual relationship between AF and anxiety disorders, in which the latter can pave a background that is favourable for the initiation and progression of the former. Anxiety is generally defined as a psychobiological emotional state or reaction that consists of unpleasant feelings of tension, apprehension, nervousness, worry, and activation of the autonomic nervous system [6]. The diagnosis of anxiety includes specific phobia, social phobia, panic disorder, agoraphobia, and generalized anxiety disorder [7]. Traditionally, anxiety has been considered a consequence of AF due to the impairment in quality of life associated with this arrhythmia. While it is well known that anxiety is an independent risk factor for cardiovascular disease, associated with a 26% increased risk of incident coronary heart disease (CHD) and a 48% increased risk of cardiac death [8–10], less is known about the role of anxiety disorders in AF onset, severity, and clinical outcomes. The recognition of the involvement of such psychological factors in the development of AF may help the identification of new clinical strategies for the management of AF.

## 2. Pathophysiological Insights for the Link between AF and Anxiety Disorders

The onset and progression of AF is the result of the interaction between three elements that form Coumel's triangle of arrhythmogenesis: the arrhythmogenic substrate, the trigger factors, and the modulation factors, of which the most common is the autonomic nervous system.

Several studies show a possible association of anxiety disorders and AF. Nevertheless, a clear relationship has never been demonstrated, this association should be based on the pathophysiological consequences of the anxious state on the neuroendocrine, coagulative, microcirculatory, and immune systems.

It is known that inflammation and oxidative stress are key players for the development of AF through atrial fibrosis, myocyte apoptosis and/or necrosis, and irregular myocellular hypertrophy with disarrangement of lines of cells and recruitment of macrophages to the endothelial surface [11]. All these factors constitute the anatomical arrhythmogenic substrate that results in shortening and dispersion of refractory period, conduction velocity slowing, and formation of reentry circuits. The arrhythmogenic substrate resulting from anatomical and electrophysiological atrial remodelling predisposes to onset and maintenance of AF.

The relationship between inflammatory cytokines and AF risk has been previously described in multiple setting, and both C-reactive protein and interleukin-6 are independently associated with AF [12–15]. Many studies showed that anxiety and depressive disorders are linked to low-grade systemic inflammation [12, 16–18]. For example, the ATTICA study evaluated various inflammation and coagulation markers among healthy adults in relation to the anxious state (assessed by Spielberger's State-Trait Anxiety Inventory, STAI). STAI score was positively correlated with C-reactive protein, interleukin-6, homocysteine, and fibrinogen levels. The ATTICA study provided strong evidence that anxiety is associated with systemic inflammation and abnormal coagulation process, possibly leading to increased cardiovascular events [6].

Moreover, stress response has been described as resulting from a “fight or flight” reaction that can be the result of endocrine, nervous, and immune systems [18].

The inflammatory state found in anxious and depressive disorders is presumably related to a hyperactivity of the hypothalamic-pituitary-adrenal (HPA) axis which is commonly seen in patients with chronic stress. Although the HPA axis in normal situations should temper inflammatory reactions, prolonged hyperactivity of the HPA axis might result in blunted anti-inflammatory responses to glucocorticoids resulting in increased inflammation [16, 19, 20] Furthermore, hyperactivity of the HPA axis with cortisol hyperproduction and corticotropin-releasing hormone (CRH) overdrive produces an imbalance of monoamines; in particular, chronic stress leads to a reduced activity of dopaminergic, serotoninergic, and noradrenergic neurons [18]. In fact, persisting hypercortisolemia decreases serotonin production. For this reason, antidepressants act on monoamines replacement as well as on modulation of cerebral glucocorticoid receptors [21, 22].

Additionally, patients suffering from anxious and depressive disorders are more likely to have increased activity of sympathetic nervous system [23] and subsequently catecholamine overload [24, 25]. It is known that elevated serum catecholamine levels can trigger Takotsubo (or stress) cardiomyopathy through microvascular endothelial damage and catecholamine cardiotoxic effects [26, 27]. On the other hand, acute emotional stress and chronic anxiety disorders are considered predisposing risk factors for stress cardiomyopathy because of the higher prevalence of these psychosocial factors than that in the acute coronary syndrome patients as well as in general population [28, 29]. In anxiety disorders, serum catecholamine levels are elevated; thus, the susceptibility to AF may be in part related to anxiety disorders through the same catecholamine-mediated myocardial injury seen in stress cardiomyopathy. Catecholamine overload in anxiety disorders could lead to the formation of the arrhythmogenic substrate and could be a trigger for the onset of paroxysmal AF. The morphological alterations caused by catecholamine overload include: extracellular matrix overproduction, contraction band necrosis, and mononuclear cell infiltration [26, 27]. Catecholamine overload leads to an extracellular accumulation of collagen alfa-1 (I) chain and to an increased ratio of collagen alfa-1 (I) chain to collagen alfa-1 (III) chain that results in a large and rapid increase in atrial fibrosis [27]. The increased release of catecholamines results in an enhanced catecholamine degradation, which in turn leads to production of reactive oxygen species [30, 31], and in increased level of profibrotic mediator like angiotensin II, TGF beta, and osteopontin [32]. On the other hand, matrix metalloproteinase are not correspondingly activated with the result of augmentation of extracellular matrix proteins, myocardial disarray, and negative atrial remodelling. Lastly, the overstimulation of beta-adrenoreceptors by supraphysiologic levels of catecholamines alters the expression of calcium-regulatory protein genes [33, 34]. The impairment of the calcium-handling system causes ultrastructural atrial remodelling and predisposes to the onset and progression of AF. Anxiety disorders can trigger AF through the increased activity of sympathetic nervous system that is known to be the most important modulation factor of Coumel's triangle of arrhythmogenesis. Patients with anxiety have reduced heart rate variability and vagal tone [35], which suggests an abnormal autonomic system regulation and represents an independent risk factor for AF [36].

Lastly, individuals who suffer from anxiety have a stimulated renin-angiotensin-aldosterone system (RAAS) [37]. Elevated levels of angiotensin II stimulate mitogen-activated protein kinases and reduce collagenase activity, which results in cardiac fibrosis and left ventricular hypertrophy. Binding of angiotensin II to angiotensin II type I receptors induces transforming growth factor-1 (TGF-1) production which promotes atrial fibrosis [27]. Thus, the hyperactivity of RAAS results in detrimental cardiac remodelling with abnormal ventricle relaxation, diastolic impairment, and increased atrial pressure and stretch. All these mechanisms can promote AF by slowing atrial conduction velocity and providing a greater atrial surface for reentry.

## 3. Discussion

As mentioned above, chronic stress and anxious state can promote AF through several mechanisms acting at different levels as trigger, modulating the autonomic nervous system and modifying the atrial substrate. In sum, anxiety disorders can interact with all the three elements of the triangle of Coumel resulting in the arrhythmogenesis of AF.

We are underlying the possible pathophysiological consequences of the chronic stress and anxious state on the neuroendocrine, coagulative, microcirculatory, and immune systems; however, a strict relationship between AF and anxiety has never been demonstrated so far. Nevertheless, several studies clearly show the strong association of anxiety symptoms and onset or recurrence of AF strengthening the hypothesis of the existence of causal link between these two disorders.

For example, Eaker et al. showed that anxiety is a risk factor for incident AF in males and females over a 10-year time period [38, 39]. Moreover, it has been reported that anxiety symptoms increased the incidence of AF after cardiac surgery and the use of beta-blockers may reduce this correlation [40, 41].

Increased sympathetic tone, lessened vagal tone, and the cardinal symptoms of anxiety could be major provocative factors of postoperative AF acting as trigger and modulating the autonomic nervous system [24, 40].

Additionally, Lange and Herrmann-Lingen [42] found that after successful electrical cardioversion, risk of recurrence of AF remains existent due to anxiety. For those AF patients who scored more than 7 on Hospital Anxiety and Depression Scale (HADS), 85% have the possibility of recurrence. In another study, Yu et al. reported an increased risk of AF recurrence due to anxiety after taking circumferential pulmonary vein ablation [24, 43]. These studies show that anxiety disorders have an impact on AF treatment success, suggesting that these psychological disturbances can have a major role on the development and progression of AF.

There is a small study which found that paroxetine reduces drug-resistant paroxysmal AF, presumably modulating vagal tone at the level of the midbrain and inhibiting the vasovagal reflex [9, 44]. In the last decades, a bulk of data demonstrated the efficacy of selective serotonin reuptake inhibitors (SSRIs) and serotonin and norepinephrine reuptake inhibitors (SNRIs) in anxiety disorders [45]. These studies may suggest the usefulness of SSRIs and SNRIs in patients with AF and anxiety disorders. Pragmatic randomized controlled trials with antidepressants are needed to explore a possible effect on the course of AF in patients with comorbid psychosocial disorders.

All these evidences suggest that anxious disorders may create an environment that is favourable of the initiation and perpetuation of AF [9]. Nevertheless, the above-mentioned studies have several limitations as such as the small sample size, the short follow-up period, and they are single-center experiences using different questionnaires with heterogeneous validity and reliability. Additionally, the rating scales used in epidemiological studies, as Eaker's one, are not necessarily the same scales used to diagnose anxiety disorders in daily clinical practice. These findings suggest that future research should involve large multicentre prospective trials, with long follow-up period using thorough, exhaustive, and homogeneous interviews and scales for the diagnoses of anxiety disorders.

The identification of anxiety disorders as independent risk factor for AF opens new scenarios in the management of this arrhythmia.

Firstly, in patients with multiple risk factors for AF, anxiety assessment should be routinely performed through standardized questionnaire, as the Hamilton Anxiety Rating Scale (HAM-A) [46], Spielberger's State Anxiety Inventory (STAI) [47], and the Zung Self-Rating Anxiety Scale (SAS) [48], to identify this psychosocial risk factor and possibly prevent AF onset and progression. Moreover, in this setting, it is important to underline the need of a clinical diagnosis of anxiety disorders in order to make a differential diagnosis with depressive disorders that might also be present as comorbidity.

Secondly, it might be interesting to evaluate whatever identification and treatment of anxious states could be useful to improve outcomes and optimize the management of this arrhythmia in patients with recurrent AF, or treatment-resistance AF.

Lastly, future prospective well-designed studies should clarify the possible causal role of anxiety disorders in the onset of AF in patients after surgery. Considering the possible causal link between AF and anxiety in patients after cardiac surgery, the usefulness of beta-blockers, benzodiazepines, and SSRI should be evaluated in reducing the incidence and in the management of postoperative AF.

## 4. Conclusions

The present review underlines the possible pathophysiological mechanisms through which anxiety disorders can promote the onset, progression, and maintenance of AF. A relationship may exist between the most common clinical arrhythmia and anxious states. The recognition of the involvement of such psychological factors in the development of AF may help the identification of new clinical strategies for the management of AF. Nevertheless, a few studies pointed out the possible role of psychological factors on the development of AF, and a clear association has not been demonstrated yet. Considering the limitations of the present studies addressing a role of anxiety disorders as risk factor for AF and the high health burden of AF, large prospective studies are necessary to elucidate this multifaceted relationship and to assess the benefits of routine anxiety assessment in AF patients and the usefulness of anxiolytics and antidepressants in the prevention and treatment of AF.
